# A novel clinical prognostic index for patients with advanced gastric cancer: possible contribution to the continuum of care

**DOI:** 10.1016/j.esmoop.2021.100234

**Published:** 2021-08-27

**Authors:** K. Shimozaki, I. Nakayama, D. Takahari, D. Kamiimabeppu, H. Osumi, T. Wakatsuki, A. Ooki, M. Ogura, E. Shinozaki, K. Chin, K. Yamaguchi

**Affiliations:** 1 Department of Gastroenterology, The Cancer Institute Hospital, Japanese Foundation for Cancer Research, Tokyo, Japan; 2 Division of Gastroenterology and Hepatology, Department of Internal Medicine, Keio University School of Medicine, Tokyo, Japan

**Keywords:** gastric or gastroesophageal junction cancer, prognostic factor, neutrophil-to-lymphocyte ratio, postprogression survival, diffuse type

## Abstract

**Background:**

The Japan Clinical Oncology Group (JCOG) prognostic index, consisting of performance status, primary tumor resected, number of metastases, and serum alkaline phosphatase, has been one of the robust prognostic indices for patients with advanced gastric cancer on the basis of which clinical trials have stratified prognosis. Only a few studies, however, have utilized the JCOG prognostic index in daily practice.

**Methods:**

We conducted a retrospective study on patients with advanced gastric cancer who received first-line platinum-containing chemotherapy at a single institute between 2011 and 2017. Prognostic factors were evaluated using a Cox proportional regression model.

**Results:**

A total of 608 patients were enrolled. Multivariate analysis showed that performance status ≥1, presence or absence of primary tumor, serum alkaline phosphatase, neutrophil-to-lymphocyte ratio ≥4, and diffuse-type histology were significantly associated with worse prognosis, whereas the number of metastases was not. Although the original prognostic index could not adequately stratify patients into three risk groups, the modified index (good: 0 and 1, moderate: 2 and 3, poor: 4-6), which was established by incorporating diffuse-type histology and high neutrophil-to-lymphocyte ratio, demonstrated excellent stratification. The median overall survival of the good (*n* = 315), moderate (*n* = 243), and poor (*n* = 54) risk groups was 20.5, 13.5, and 10.2 months, respectively. Hazard ratios (HRs) were 1.69 [95% confidence interval (CI), 1.40-2.04; good versus moderate] and 1.52 (95% CI, 1.11-2.08; moderate versus poor). This novel index also demonstrated a statistically significant stratification of survival after progression following first-line chemotherapy (good versus moderate: HR, 1.41; 95% CI, 1.16-1.70; moderate versus poor: HR, 2.00; 95% CI, 1.45-2.74).

**Conclusions:**

The modified JCOG prognostic index showed excellent stratification of overall survival in real-world patients, which could also help determine the need for treatment changes throughout the continuum of chemotherapy.

## Highlights

• The applicability of the JCOG prognostic index in daily practice was investigated for advanced gastric cancer (AGC).
• The JCOG prognostic index could not adequately stratify patients into three risk groups.
• The modified JCOG prognostic index incorporates diffuse-type histology and high NLR (≥4) into the JCOG prognostic index.
• The modified JCOG prognostic index showed excellent stratification in terms of overall survival and progression.
• The modified JCOG prognostic index may help determine the need for treatment changes during first-line chemotherapy in AGC.

## Introduction

Advanced gastric cancer (AGC) is one of the most common malignancies and the third leading cause of cancer-related death worldwide.^1^ Palliative chemotherapy remains the standard of care for patients with metastatic gastric cancer, with the combination of fluoropyrimidine and platinum analog having been recognized as the standard first-line treatment.^2^ Over the past few years, therapeutic options for second- or later-line treatment have improved the prognosis for AGC. Notably, ramucirumab, an anti-vascular endothelial growth factor (VEGF) antibody, has significantly improved overall survival (OS) in combination with paclitaxel for second-line treatment.^3^ For third-line treatment options, the anti-programmed cell death protein 1 (PD-1) antibody nivolumab and trifluridine/tipiracil have shown greater efficacy compared with placebo.^4^^,^^5^ The median survival time of patients with AGC, however, has remained at ∼15 months,^6^^,^^7^ with most patients failing to exhaust all treatment options.^8^ Thus, further improving the prognosis of patients with AGC is strongly warranted.

Several studies have repeatedly identified poor prognostic factors, including poor Eastern Cooperative Oncology Group (ECOG) performance status (PS), not receiving gastrectomy, presence of peritoneal metastases, and blood test abnormalities [e.g. high alkaline phosphatase (ALP), low albumin, high lactate dehydrogenase, or high neutrophil-to-lymphocyte ratio (NLR)], across different cohorts of patients with AGC.^9^, ^10^, ^11^, ^12^, ^13^, ^14^ Stratification models based on prognostic factors have been suggested in AGC and other malignancies.^15^^,^^16^ In Japan, Takahari et al.^17^ reported that PS ≥1, number of metastatic sites ≥2, no prior gastrectomy, and elevated ALP were associated with poor prognosis and proposed the Japan Clinical Oncology Group (JCOG) prognostic index based on these factors and by analyzing individual data from 760 patients who participated in the JCOG 9912 trial. Furthermore, a number of studies have confirmed the ability of the JCOG prognostic index to stratify the prognosis of patients included in the SPIRITS and G-SOX trials.^18^, ^19^, ^20^ The clinical application of the aforementioned index, however, has yet to be comprehensively investigated.^21^^,^^22^ Several prognostic models, including the JCOG prognostic index, have been originally developed to recruit appropriate patient populations into clinical trials.^9^ For instance, the actual number of patients having diffuse-type histology of AGC might be higher compared with the number of patients enrolled in the clinical trials.^10^, ^11^, ^12^ Considering that peritoneal dissemination may cause massive ascites, inadequate oral intake, and bowel obstruction, some patients with diffuse-type AGC might have been excluded from clinical trials owing to concerns about poor prognosis. As such, the current study sought to validate the clinical utility of a refined prognostic index and explore the potential value of these prognostic factors in guiding treatment strategies for daily practice.

## Materials and methods

### Patients

The present study was retrospectively conducted between January 2011 and December 2017 at the Cancer Institute Hospital of the Japanese Foundation for Cancer Research (JFCR). The following inclusion criteria were used to select patients for the present study: (i) unresectable or metastatic gastric or gastroesophageal junction cancer, (ii) histologically or cytologically confirmed adenocarcinoma, and (iii) platinum-based chemotherapy as first-line treatment. The exclusion criteria were as follows: (i) disease relapse during or within 6 months after adjuvant or neoadjuvant therapy, (ii) chemotherapy with immune checkpoint blockade, (iii) adjuvant chemotherapy after R0 metastasectomy, (iv) initiated on first-line chemotherapy at another hospital, or (v) known other advanced cancer.

The current study was approved by the Institutional Review Board of the Cancer Institute Hospital of JFCR (Tokyo, Japan; approval date: 11 November 2020; registry number: 20201206). Given the retrospective nature of the study, informed consent was waived with the opportunity to opt out from the research. This study was conducted in accordance with the Helsinki Declaration.

### Definition of the JCOG prognostic index

The JCOG prognostic index is based on the following factors: PS ≥1, number of metastatic sites ≥2, no prior gastrectomy, and elevated ALP, with patients having 0-1, 2-3, and 4 factors being defined as having good, moderate, and poor risk, respectively.

### Statistical analyses

NLR was determined by dividing the neutrophil count by the lymphocyte count, with a cut-off value of ≥4 being used based on previous studies. OS was defined as the duration from first-line chemotherapy initiation to death from any cause. Progression-free survival (PFS) was defined as the duration from first-line chemotherapy initiation to disease progression or death from any cause. Postprogression survival (PPS) was defined as the duration for which patients survived following progressive disease during first-line treatment. OS, PFS, and PPS were calculated using the Kaplan–Meier method. The Cox proportional hazard regression model was used to calculate hazard ratios (HRs). Comparisons between groups were conducted using the Student's *t*-test and Pearson's chi-square test for continuous and categorical variables, respectively. Univariate and multivariate analyses of survival were carried out using the Cox proportional regression model. All *P* values were based on a two-sided hypothesis, with those <0.05 being considered statistically significant. All statistical analyses were carried out using the JMP version 14.2.0 software (SAS Institute, Cary, NC).

## Results

### Patient characteristics

**Figure 1:**
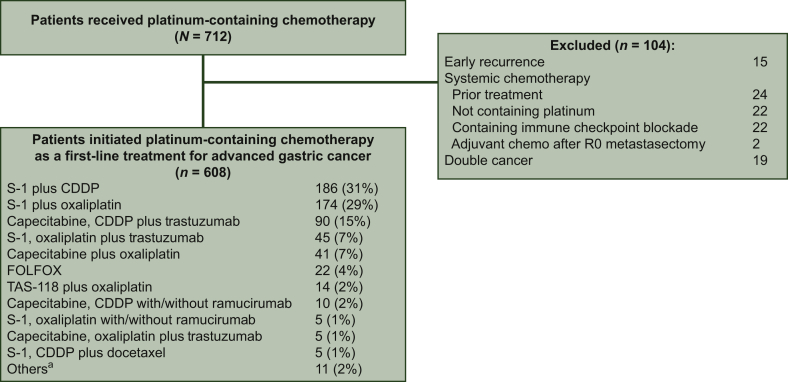
Patient flow chart. CDDP, cisplatin; 5-FU, fluorourcil. ^a^ 5-FU plus CDDP plus trastuzumab (*n* = 4); S-1 plus oxaliplatin plus andecaliximab (*n* = 4); capecitabine plus CDDP (*n* = 2); S-1 plus CDDP plus andecaliximab (*n* = 1).

Among the 712 consecutive patients who received platinum-containing chemotherapy at the JFCR between January 2011 and December 2017, 608 were eligible for inclusion (Figure 1). Patient's characteristics are described in Table 1. A total of 197 (32%) patients received gastrectomy before chemotherapy, with 374 (62%), 214 (35%), and 20 (3%) having an ECOG PS of 0, 1, and ≥2, respectively. Moreover, 168 (28%) patients had ≥2 metastatic sites; 153 (25%) had an ALP ≥upper limit of normal (ULN) upon chemotherapy initiation, and 398 (65%) had a diffuse-type Lauren classification.

**Table 1:** Patient characteristics

Characteristics (*N* = 608)		
Age (years), median (range)		63 (21-84)
Sex, male, *n* (%)		376 (62)
Disease status, *n* (%)	Recurrent	123 (20)
	Metastatic	470 (77)
	Unresectable	15 (2)
Prior gastrectomy, *n* (%)	Yes	197 (32)
	No	411 (68)
Primary tumor site, *n* (%)	EGJ	125 (21)
	Stomach	474 (79)
Histological type, *n* (%)	Intestinal	207 (34)
	Diffuse	398 (65)
	Unknown	3 (1)
HER2, *n* (%)	Positive	147 (24)
	Negative	442 (73)
	Unknown	19 (3)
ECOG PS, *n* (%)	0	374 (62)
	1	214 (35)
	≥2	20 (3)
Metastatic site, *n* (%)	Peritoneum	269 (44)
	Liver	159 (26)
	Lung	32 (5)
	Lymph node	189 (30)
	Bone	33 (5)
No. of metastatic sites, *n* (%)	0-1	440 (72)
	≥2	168 (28)
Adjuvant chemotherapy, *n* (%)	Yes	91 (16)
LDH, U/l median (range)		189 (104-3778)
ALP, U/l median (range)		238.5 (12.5-7724)
NLR, median (range)		2.94 (0.48-47.0)
CEA, ng/ml median (range)		3.5 (0-50 000)
CA19-9, U/ml median (range)		16.3 (1.1-50 000)

ALP, alkaline phosphatase; CEA, carcinoembryonic antigen; ECOG, Eastern Cooperative Oncology Group; EGJ, esophagogastric junction; HER2, human epidermal growth factor receptor 2; LDH, lactate dehydrogenase; NLR, neutrophil-to-lymphocyte ratio; PS, performance status.

At the cut-off period for data collection in February 2021, the median follow-up period was 15.1 months, with 552 (91%) patients having progressed after first-line treatment and 494 (81%) succumbing to their disease. The median OS, PFS, and PPS, was 16.3 months [95% confidence interval (CI), 14.9-17.9 months], 7.1 months (95% CI, 6.5-7.8 months), 7.2 months (95% CI, 6.6-8.2 months), respectively (Supplementary Figure S1, available at https://doi.org/10.1016/j.esmoop.2021.100234).

### Survival according to the JCOG prognostic index

**Figure 2:**
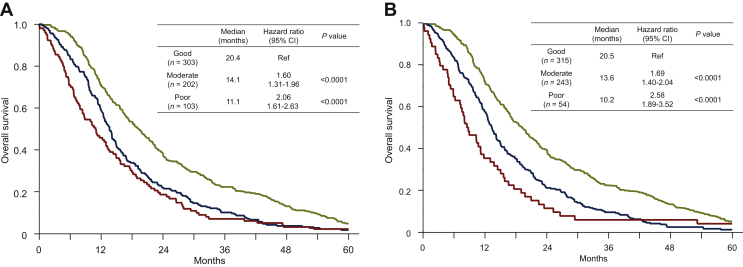
Kaplan–Meier estimates of overall survival according to the Japan Clinical Oncology Group (JCOG) prognostic index (A) and the JCOG prognostic index plus diffuse type and NLR ≥4 (modified JCOG prognostic index) (B). CI, confidence interval; NLR, neutrophil-to-lymphocyte ratio; Ref, reference.

Figure 2A presents the OS classified according to the JCOG prognostic index. After applying the JCOG prognostic index, the good (*n* = 303), moderate (*n* = 202), and poor (*n* = 103) risk groups exhibited a median OS of 20.4, 14.1, and 11.1 months, respectively, with the moderate and poor risk groups having an HR of 1.60 (95% CI, 1.31-1.96; *P* < 0.0001) and 2.06 (95% CI, 1.61-2.63; *P* < 0.0001) for OS relative to the good risk group, respectively. No significant difference in OS was observed between the moderate and poor risk groups (HR, 1.28; 95% CI, 0.99-1.65; *P* = 0.0527). The JCOG prognostic index did not adequately stratify patients in actual clinical practice.

### Univariate and multivariate Cox regression analyses for OS

Table 2 summarizes the results of univariate and multivariate Cox regression analyses for OS using baseline characteristics and laboratory tests. The following factors were independently associated with poor prognosis in this cohort: PS ≥1 (HR, 1.55; 95% CI, 1.29-1.87; *P* < 0.001), diffuse-type histology (HR, 1.53; 95% CI, 1.29-1.84; *P* < 0.001), human epidermal growth factor receptor 2 (HER2) negativity (HR, 1.46; 95% CI, 1.14-1.83; *P* = 0.002), no prior gastrectomy (HR, 1.30; 95% CI, 1.07-1.58; *P* = 0.007), ALP ≥ ULN (HR, 1.27; 95% CI, 1.02-1.57; *P* = 0.027), NLR ≥4 (HR, 1.23; 95% CI, 1.01-1.50; *P* = 0.034), respectively. Contrary to the JCOG prognostic index, metastatic sites ≥2 was not significantly associated with poor prognosis (HR, 1.14; 95% CI, 0.93-1.39; *P* = 0.19).

**Table 2:** Univariate and multivariate analyses of survival

Variables	Category (Ref)	Univariate			Multivariate		
		HR	95% CI	*P* value	HR	95% CI	*P* value
Age, years	<65 versus ≥65 (Ref)	1.09	0.92-1.31	0.29			
Sex	Female versus male	1.08	0.90-1.29	0.40			
ECOG PS	≥1 versus 0	1.68	1.40-2.01	<0.0001∗	1.55	1.29-1.87	<0.0001∗
Histological type	Diffuse versus intestinal	1.50	1.24-1.82	<0.0001∗	1.53	1.29-1.84	<0.0001∗
Tumor status	Metastatic/unresectable versus Recurrent	1.38	1.09-1.75	0.0065∗			
HER2	Negative versus positive	1.35	1.10-1.67	0.0043∗	1.46	1.14-1.83	0.002∗
Gastrectomy	No versus Yes	1.36	1.12-1.65	0.0014∗	1.30	1.07-1.58	0.007∗
No. of metastatic sites	≥2 versus 0-1	1.26	1.03-1.53	0.019∗	1.14	0.93-1.39	0.19
Peritoneum	Yes versus No	1.41	1.18-1.68	0.0001∗			
Liver	Yes versus No	1.00	0.81-1.22	0.98			
Lymph node	Yes versus No	1.11	0.91-1.35	0.29			
ALP	≥ULN versus <ULN	1.38	1.13-1.69	0.0013∗	1.27	1.02-1.57	0.027∗
NLR	≥4 versus <4	1.45	1.21-1.75	<0.0001∗	1.23	1.01-1.50	0.034∗

ALP, alkaline phosphatase; CI, confidence interval; ECOG, Eastern Cooperative Oncology Group; HER2, human epidermal growth factor receptor 2; HR, hazard ratio; NLR, neutrophil-to-lymphocyte ratio; PS, performance status; Ref, reference; ULN, upper limit of normal.

∗ *P* < 0.05.

### Modification of the JCOG prognostic index and prognosis

Multivariate analysis revealed six independent prognostic factors (PS ≥1, no prior gastrectomy, ALP ≥ ULN, diffuse-type histology, HER2 negativity, and NLR ≥4), three of which overlapped with the original JCOG prognostic index. Therefore, we herein modified the JCOG prognostic index in an attempt to make it more suitable for patient populations in actual clinical practice. Accordingly, two factors (diffuse-type histology and high NLR level) were added to the original JCOG prognostic index. Thereafter, the new index, namely the modified JCOG prognostic index, was used to classify patients into good (0 to 2 factors, *n* = 315), moderate (3 or 4 factors, *n* = 243), and poor (5 or 6 factors, *n* = 54) risk groups, which had a median OS of 20.5, 13.5, and 10.2 months, respectively. Moreover, the moderate and poor risk groups had an HR of 1.69 (95% CI, 1.40-2.04; *P* < 0.0001) and 2.58 (95% CI, 1.89-3.52; *P* < 0.0001) for OS relative to the good risk group, respectively (Figure 2B). The poor risk group also had significantly worse prognosis compared with the moderate risk group (HR, 1.52; 95% CI, 1.11-2.08; *P* = 0.0085). Overall, the modified JCOG prognostic index showed excellent stratification of patients according to prognosis, with significant differences between each group.

Survival according to HER2 status was also evaluated using the modified JCOG clinical prognostic index after excluding those whose HER2 status was unknown or not evaluated (*n* = 19). Notably, the modified JCOG prognostic index showed excellent stratification according to survival regardless of HER2 status (Supplementary Figure S2A and B, available at https://doi.org/10.1016/j.esmoop.2021.100234).

### PFS and PPS according to the modified JCOG prognostic index

According to the modified JCOG prognostic index, the good, moderate, and poor risk groups had a median PFS of 8.2, 5.9, and 4.6 months, respectively, Moreover, the moderate and poor risk group had an HR of 1.55 (95% CI, 1.29-1.85; *P* < 0.0001) and 1.78 (95% CI, 1.31-2.41; *P* = 0.0002) for PFS, respectively (Supplementary Figure S3, available at https://doi.org/10.1016/j.esmoop.2021.100234). Notably, no significant difference was found between the moderate and poor risk groups (HR, 1.14; 95% CI, 0.84-1.56; *P* = 0.37). Overall, the modified JCOG prognostic index was unable to adequately stratify patients according to PFS.

**Figure 3:**
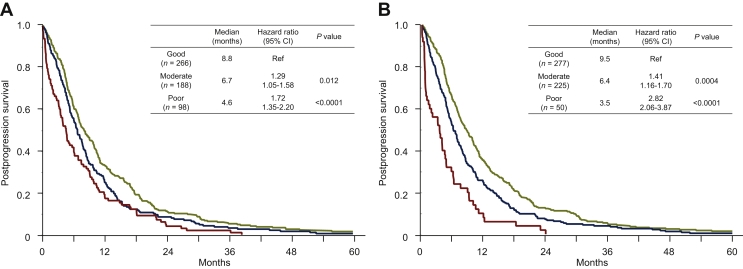
Kaplan–Meier estimates of postprogression survival according to the Japan Clinical Oncology Group (JCOG) prognostic index (A) and the modified JCOG prognostic index (B). CI, confidence interval; Ref, reference.

Interestingly, the modified JCOG prognostic index clearly showed the better stratification compared with the JCOG prognostic index according to survival time after progression following first-line chemotherapy in our cohort (Figure 3A), with the good (*n* = 277), moderate (*n* = 225), and poor (*n* = 50) risk groups having a median PPS of 9.5, 6.4, and 3.5 months, respectively. Moreover, the moderate and poor risk groups had an HR of 1.41 (95% CI, 1.16-1.70; *P* = 0.0004) and 2.82 (95% CI, 2.06-3.87; *P* < 0.0001) for PPS relative to the good risk group, respectively (Figure 3B). The poor risk group also had a significantly worse PPS compared with the moderate risk group (HR, 2.00; 95% CI, 1.45-2.74; *P* < 0.0001).

## Discussion

The present study validated the clinical utility of the JCOG prognostic index using data obtained from an adequate number of actual clinical patients. Overall, the current study showed that the JCOG prognostic index could not adequately stratify real-world patients with AGC according to prognosis, perhaps due to the greater number of patients with diffuse-type AGC included in the present study. Furthermore, 44% had peritoneal metastases compared with the 32% rate in the cohort that was evaluated during the establishment of the original JCOG prognostic index. Interestingly, multivariate analysis of our cohort showed that the number of metastases was not significantly associated with poor prognosis. Certainly, some patients with diffuse-type AGC only have peritoneal dissemination without any other organ involvement,^23^, ^24^, ^25^ indicating that the number of metastatic sites does not adequately reflect the prognosis of patients in daily practice. Therefore, the original JCOG prognostic index alone may not clearly stratify actual clinical patients, especially those with poor prognosis.

We herein proposed a novel prognostic index, namely the modified JCOG prognostic index, which incorporates histological type and NLR level, herein identified as independent prognostic factors, into the original JCOG prognostic index. Compared with the original index, the modified JCOG prognostic index was able to better stratify patients with AGC in clinical practice according to prognosis. As previously reported, patients with diffuse-type histology had extremely worse prognosis compared with those with intestinal-type gastric cancer.^26^ Moreover, high NLR levels have been reported to predict worse prognosis in AGC.^14^^,^^27^^,^^28^ Several studies have suggested that NLR levels indicate cancer-associated inflammatory response, lymphocyte-mediated antitumor response, and production of cytokines, including tumor necrosis factor, interleukin-1, interleukin-6, and angiogenic factor VEGF.^29^^,^^30^ Hence, NLR levels take into account a host’s immunological response when stratifying patients with AGC according to prognosis. Unsurprisingly, the aforementioned have been confirmed to be prognostic factors in previous studies, suggesting reproducibility. We excluded the parameters of tumor status and the presence of peritoneal metastases in the multivariate analysis, although these were identified to be significantly associated with poor prognosis in the univariate analysis; the tumor status overlapped with the presence or absence of primary tumor (82% of patients diagnosed with recurrent AGC had undergone prior gastrectomy), and the presence of peritoneal metastases was correlated with diffuse-type histology (77% of patients with peritoneal metastases had diffuse-type histology). Overall, the modified JCOG prognostic index demonstrated excellent stratification of AGC prognosis by more comprehensively considering tumor status and patients' conditions compared with the original index.

Notably, the modified JCOG prognostic index also excellently stratified patient prognosis after disease progression following first-line treatment. In AGC, PPS has been reported to better correlate with OS than with PFS, suggesting the increasing significance of sequential treatment after fist-line treatment for prolonging of the survival of patients with AGC.^31^, ^32^, ^33^ Fortunately, the therapeutic options for AGC after progression following first-line chemotherapy have been increasing.^3^, ^4^, ^5^ Thus, providing subsequent chemotherapies after first-line treatment at the proper timing is certainly important. Opportunities for providing subsequent treatment of certain patients, however, such as those with peritoneal dissemination, are often missed given the difficulty of accurately determining the timing of disease progression with existing diagnostic modalities, such as computed tomography or serum tumor markers. In fact, our findings showed that patients in the poor risk group received subsequent chemotherapy less frequently (58%) than those in the good (80%) or moderate (76%) risk group (data not shown). We presume that patients categorized into the poor risk group could not receive subsequent chemotherapy at the proper timing due to the difficulty of evaluating disease progression. Iwasa et al.^34^ proposed practical guidelines for comprehensively evaluating disease progression using prognostic factors, as well as radiographic imaging, cancer-related symptoms, and tumor markers. Thus, prognostic factors for evaluating disease progression should be considered more carefully in order to change treatment more appropriately. Furthermore, we believe that the modified JCOG prognostic index could help clinicians not only predict the prognosis of patients with AGC, but also maximize the benefits of subsequent chemotherapy.

Of note, the modified JCOG prognostic index showed that the poor risk group had a median OS of >10 months, which seemed relatively more favorable compared with those of poor risk groups included in previous reports.^9^^,^^17^^,^^18^ Differences in the prognosis between HER2-positive and -negative patients could have affected such results. Following the success of the ToGA trial, several studies have reported a favorable prognosis for HER2-positive AGC.^35^^,^^36^ Indeed, the current study has identified HER2-negative status as an independent negative prognostic factor. Among the patients included in the poor risk group, those with HER2-positive and -negative status had a median OS of >11 months and ∼8 months, respectively, which was nearly half of the median OS and similar to the median PFS of our entire cohort. Notably, the modified JCOG prognostic index was able to excellently stratify patient prognosis, regardless of HER2 status.

Some limitations inherent to the current study's retrospective nature are worth noting. First, platinum-containing treatment tended to be prescribed for patients with more favorable conditions, which might have favorably affected the survival of the whole cohort. However, given that platinum-doublet chemotherapy has been recognized as the standard of care for AGC, the majority of patients in clinical practice receive platinum-containing treatment, with those receiving monotherapy having characteristics different from those receiving platinum-doublet chemotherapy. Second, the current study retrospectively analyzed a cohort of patients from a single specialized cancer hospital, which might have introduced selection bias considering the decreased tendency of including patients with serious underlying diseases or multiple comorbidities. Moreover, the characteristics of our patients might have differed from those in community hospitals. Third, although the proposed prognostic index was constructed from the cohort that had a sample size comparable with that in previous studies and has yet to be validated in other cohorts, it was constructed based on the JCOG prognostic index, which had been validated in another cohort. Furthermore, the prognostic factors that were identified in this study, such as histological type and NLR level, have also been reported as prognostic factors for AGC in previous studies. Therefore, we believe that the robustness and reproducibility of the modified JCOG prognostic index might have been partially addressed. We plan to perform validation of the proposed prognostic index in a multi-institutional study with a large number of patients.

In conclusion, the JCOG prognostic index alone did not adequately stratify actual clinical patients with AGC according to survival. However, the modified JCOG prognostic index, which incorporated diffuse-type histology and high NLR level into the JCOG prognostic index, was able to excellently stratify prognosis not only upon first-line chemotherapy initiation, but also after progression. Considering that the modified JCOG prognostic index could potentially help guide treatment changes at the appropriate timing, further validation studies of this novel prognostic index are warranted in the near future.

## Supplementary data

Supplementary Figure S1 Kaplan–Meier estimates of overall survival (A), progression-free survival (B), and postprogression survival (C) CI, confidence interval.

Supplementary Figure S2 Kaplan–Meier estimates of overall survival according to the modified JCOG prognostic index in HER2-positive (A) and -negative (B) gastric cancer.

Supplementary Figure S3 Kaplan–Meier estimates of progression-free survival according to the modified JCOG prognostic index.
