# Supplementary material for: A novel clinical prognostic index for patients with advanced gastric cancer: possible contribution to the continuum of care
Source: ESMO Open. 2021 Aug 27;6(5):100234. doi: 10.1016/j.esmoop.2021.100234 (PMC8405892; doi:10.1016/j.esmoop.2021.100234)
Supplement: Supplementary Figure S1 — Kaplan–Meier estimates of overall survival (A), progression-free survival (B), and postprogression survival (C) CI, confidence interval. [file mmc1.pptx]

## Slide 1
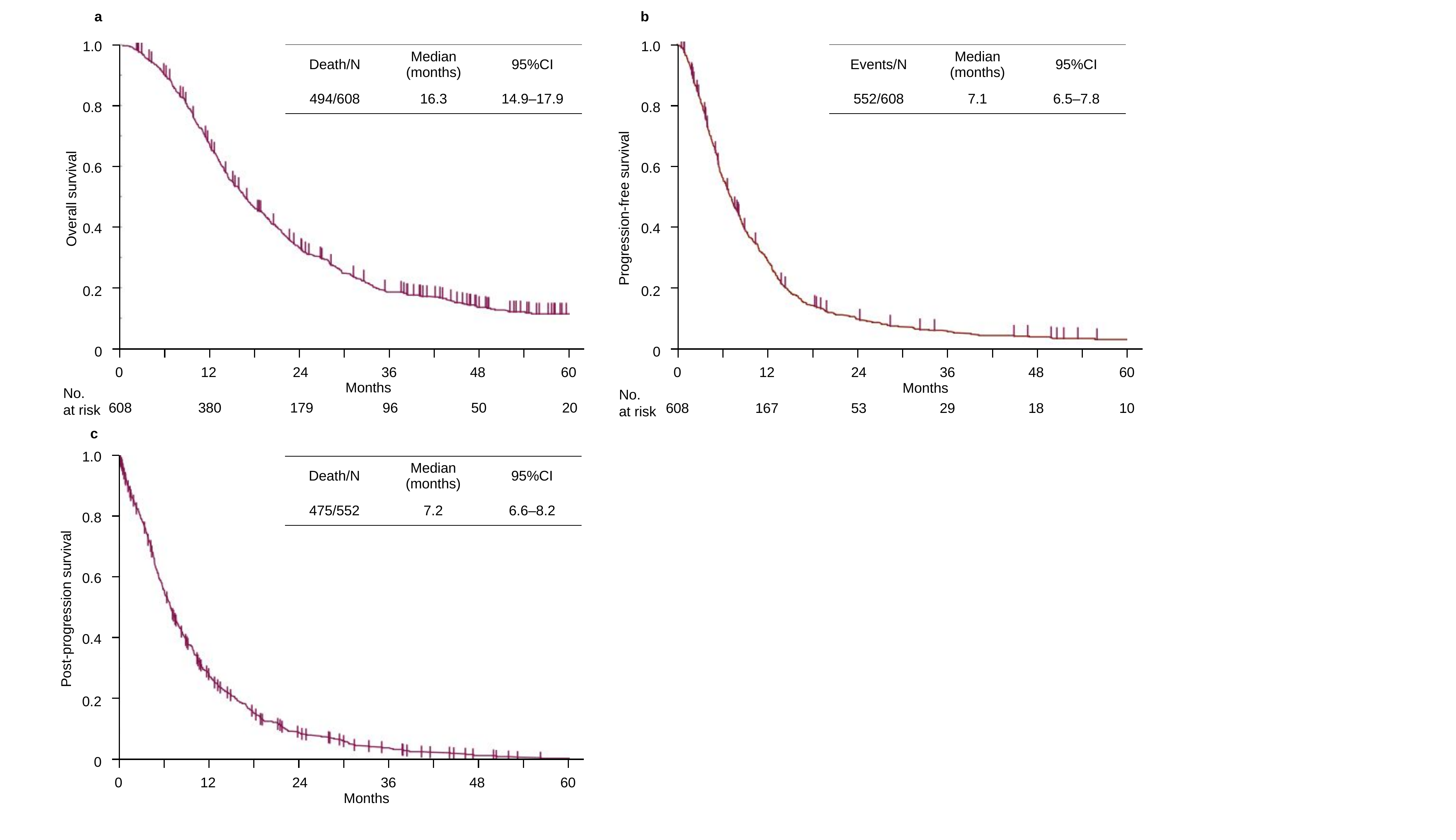

a
b
1.0
60
0
12
24
36
48
0.8
0.6
0.4
0.2
0
1.0
60
0
12
24
36
48
0.8
0.6
0.4
0.2
0
| Events/N | Median (months) | 95%CI |
| --- | --- | --- |
| 552/608 | 7.1 | 6.5–7.8 |
| Death/N | Median (months) | 95%CI |
| --- | --- | --- |
| 494/608 | 16.3 | 14.9–17.9 |
Overall survival
Progression-free survival
Months
Months
No.
at risk
No.
at risk
608
380
179
96
50
20
608
167
53
29
18
10
c
1.0
60
0
12
24
36
48
0.8
0.6
0.4
0.2
0
| Death/N | Median (months) | 95%CI |
| --- | --- | --- |
| 475/552 | 7.2 | 6.6–8.2 |
Post-progression survival
Months
