# Supplementary material for: A novel clinical prognostic index for patients with advanced gastric cancer: possible contribution to the continuum of care
Source: ESMO Open. 2021 Aug 27;6(5):100234. doi: 10.1016/j.esmoop.2021.100234 (PMC8405892; doi:10.1016/j.esmoop.2021.100234)
Supplement: Supplementary Figure S2 — Kaplan–Meier estimates of overall survival according to the modified JCOG prognostic index in HER2-positive (A) and -negative (B) gastric cancer. [file mmc2.pptx]

## Slide 1
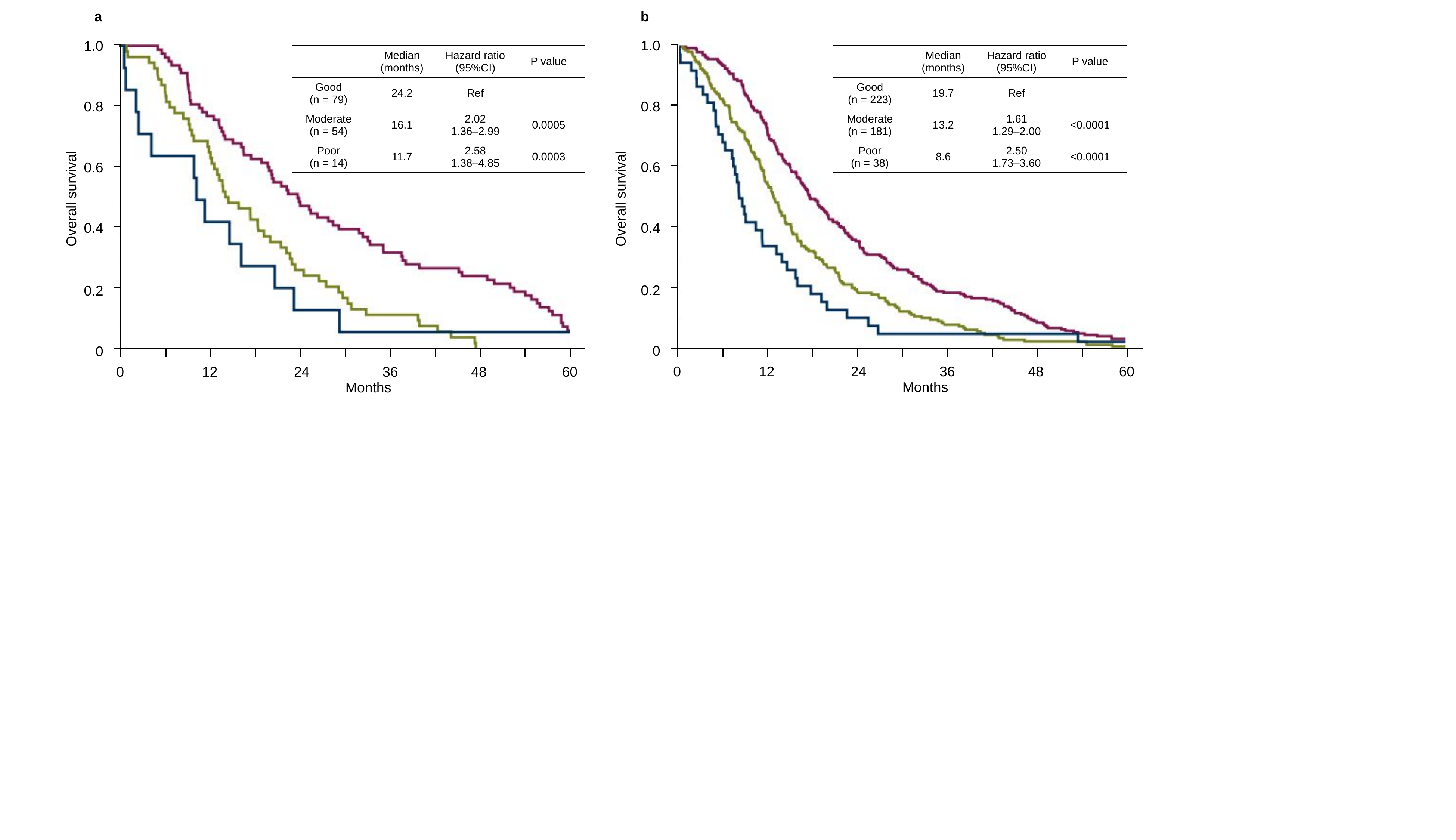

a
b
1.0
60
0
12
24
36
48
0.8
0.6
0.4
0.2
0
1.0
60
0
12
24
36
48
0.8
0.6
0.4
0.2
0
| | Median (months) | Hazard ratio (95%CI) | P value |
| --- | --- | --- | --- |
| Good (n = 79) | 24.2 | Ref | |
| Moderate (n = 54) | 16.1 | 2.02 1.36–2.99 | 0.0005 |
| Poor (n = 14) | 11.7 | 2.58 1.38–4.85 | 0.0003 |
| | Median (months) | Hazard ratio (95%CI) | P value |
| --- | --- | --- | --- |
| Good (n = 223) | 19.7 | Ref | |
| Moderate (n = 181) | 13.2 | 1.61 1.29–2.00 | <0.0001 |
| Poor (n = 38) | 8.6 | 2.50 1.73–3.60 | <0.0001 |
Overall survival
Overall survival
Months
Months
