# Supplementary material for: A novel clinical prognostic index for patients with advanced gastric cancer: possible contribution to the continuum of care
Source: ESMO Open. 2021 Aug 27;6(5):100234. doi: 10.1016/j.esmoop.2021.100234 (PMC8405892; doi:10.1016/j.esmoop.2021.100234)
Supplement: Supplementary Figure S3 — Kaplan–Meier estimates of progression-free survival according to the modified JCOG prognostic index. [file mmc3.pptx]

## Slide 1
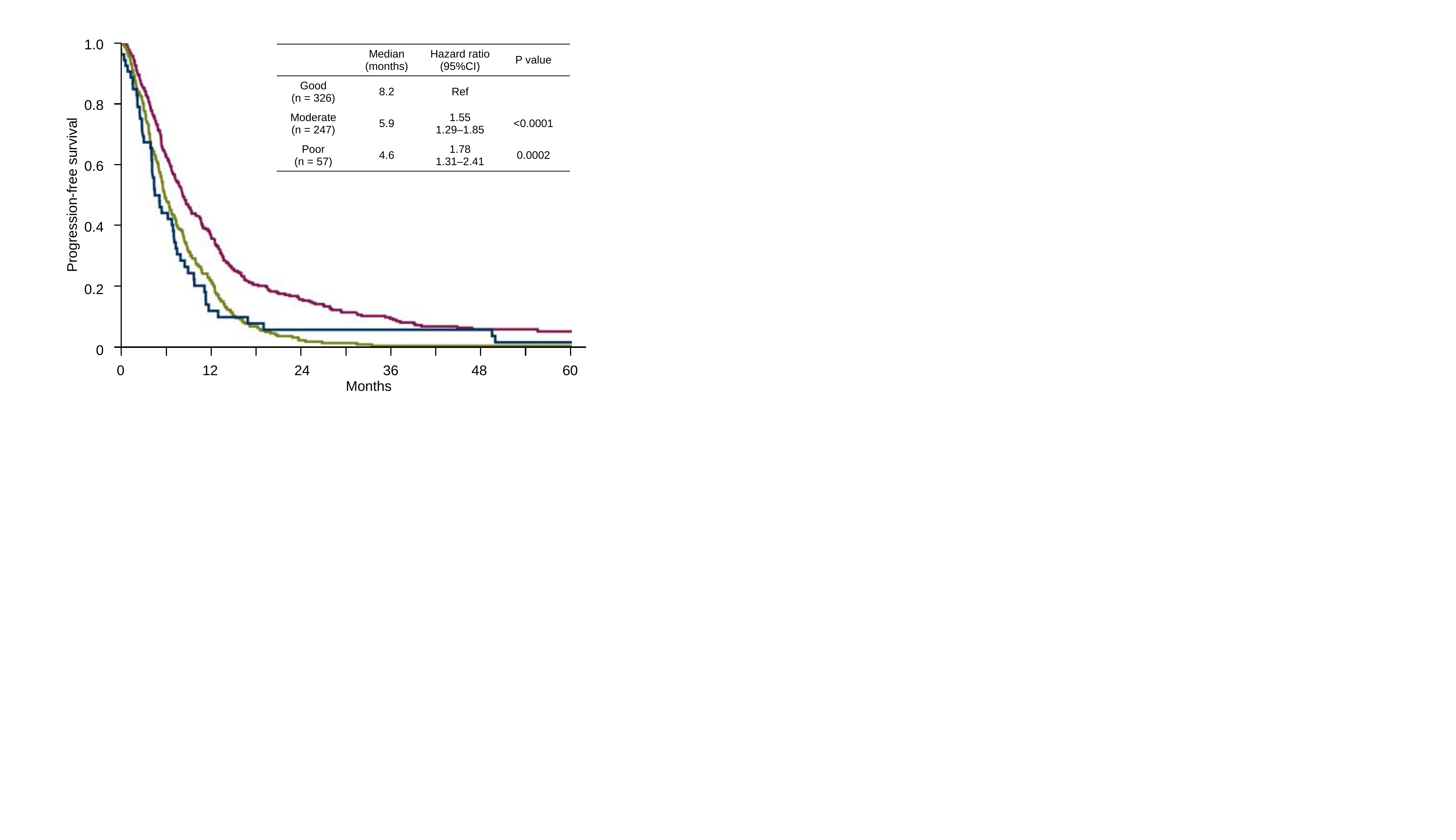

1.0
60
0
12
24
36
48
0.8
0.6
0.4
0.2
0
| | Median (months) | Hazard ratio (95%CI) | P value |
| --- | --- | --- | --- |
| Good (n = 326) | 8.2 | Ref | |
| Moderate (n = 247) | 5.9 | 1.55 1.29–1.85 | <0.0001 |
| Poor (n = 57) | 4.6 | 1.78 1.31–2.41 | 0.0002 |
Progression-free survival
Months
